# Self-supporting Co_3_O_4_/Graphene Hybrid Films as Binder-free Anode Materials for Lithium Ion Batteries

**DOI:** 10.1038/s41598-018-21436-4

**Published:** 2018-02-16

**Authors:** Shouling Wang, Ronghua Wang, Jie Chang, Ning Hu, Chaohe Xu

**Affiliations:** 1 College of Materials Science and Engineering, Chongqing University, Chongqing, 400044 China; 2 College of Aerospace Engineering, and The State Key Laboratory of Mechanical Transmissions, Chongqing University, Chongqing, 400044 China; 3 School of Chemistry and Materials Engineering, Chizhou University, Chizhou, 247000 China; 4 Key Laboratory of Low-grade Energy Utilization Technologies and Systems of the Ministry of Education of China, Chongqing, 400044 China

## Abstract

A self-supporting Co_3_O_4_/graphene hybrid film has been constructed via vacuum filtration of Co(OH)_2_ nanosheet and graphene, followed by a two-step thermal treatment. Within the hybrid film, Co_3_O_4_ nanoparticles with size of 40~60 nm uniformly *in-situ* grew on the surface of graphene, forming a novel porous and interleaved structure with strong interactions between Co_3_O_4_ nanoparticles and graphene. Such fascinating microstructures can greatly facilitate interfacial electron transportation and accommodate the volume changes upon Li ions insertion and extraction. Consequently, the binder-less hybrid film demonstrated extremely high reversible capacity (1287.7 mAh g^*−*1^ at 0.2 A g^*−*1^), excellent cycling stability and rate capability (1110 and 800 mAh g^*−*1^ at 0.5 and 1.0 A g^*−*1^, respectively).

## Introduction

The increasing demand for high performance energy storage applications makes it urgent to develop new lithium ion batteries (LIBs) which possess enhanced capacity, cyclic stability and rate capability. Transitional metal oxides (TMOs), have been considered as prominent anode materials, thanks to their high theoretical specific capacity. In this context, various anode materials with different novel structures, such as Co_3_O_4_, Fe_2_O_3_, SnO_2_ and TiO_2_^1–3^, have dramatically been explored. Among these materials, Co_3_O_4_ is especially noticeable due to its ease of synthesis and relative large theoretical specific capacity (~890 mAh g^−1^)^4–6^. Nevertheless, the practical application of this material is limited by its extremely low electrical conductivity as well as the rapid capacity attenuation resulted from the volume expansion/contraction as well as materials pulverization during cycling. One effective strategy to solve these problems is to design hybrid anode materials, which can not only provide active sites but also accommodate the volume change during charging/discharging process, thus attributes to higher capacity and cycling stability.

Graphene sheets (GS), with high conductivity, excellent mechanical flexibility and ultra-high specific surface area (~2360 m^2 ^g^−1^), is envisioned as excellent candidates for substrate materials. The unique structure as well as distinctive electrical and mechanical performance is favorable to endure the strain caused by volume change and provide a continuous electron conducting networks, thus could greatly improve the electrochemical performance of active materials^7–9^. Recently, much effort has been conducted on integration of Co_3_O_4_ with graphene as anode materials for LIBs^10,11^. For example, Co_3_O_4_/graphene nanoflowers has been synthesized through a two-step process^12^, involving a hydrothermal reaction of Co(NO_3_)_2_·6H_2_O and CTAB with graphene oxide (GO) followed by a calcination. The reversible capacity of the as-synthesized composites is as high as 1120.8 mAh g^−1^ at 1 A g^−1^. Wu *et al*. employed a surfactant-assisted hydrothermal route to synthesize Co_3_O_4_/nitrogen-doped graphene, within which Co_3_O_4_ nanoparticles with size of ~15 nm homogeneous distributed in the macropore-walls formed by graphene. The obtained composites showed excellent electrochemical performance^13^. Yang *et al*. reported the preparation of porous Co_3_O_4_ nanofibers coated with graphene layer, which delivers a capacity of 900 mAh g^−1^ at 1A g^−1^^14^. These results demonstrated the integration of graphene with Co_3_O_4_ can remarkably improve the electrical conductivity and alleviate the volume changes of Co_3_O_4_ during charge/discharge reactions, thus significantly enhanced the electrochemical performance. Even though, most Co_3_O_4_/graphene composites are powder materials, which need a complex slurry coating process in the conventional electrode preparation method. Moreover, the electrochemical-inactive polymer binder and carbon black are required to prepare electrodes, which would increase the extra weight and decrease the energy density of the actual battery^15,16^.

Herein, we report the synthesis of a self-supporting Co_3_O_4_/graphene hybrid film, which was directly used as anode materials for LIBs without any binder or additives. Co_3_O_4_ nanoparticles (40~60 nm) *in-situ* grew on the surface of GS, forming a novel interleaved structure with strong interfacial interactions. The hybrid electrode showed extremely high reversible capacity (1287.7 mAh g^−1^ at 0.2 A g^−1^), good cycling stability (capacity retention of 85.5% after 100 cycles) and excellent rate capability, owing to the fascinating microstructure and binder-free electrode nature, demonstrating great potential to be used in energy storage filed.

## Results

**Figure 1:**
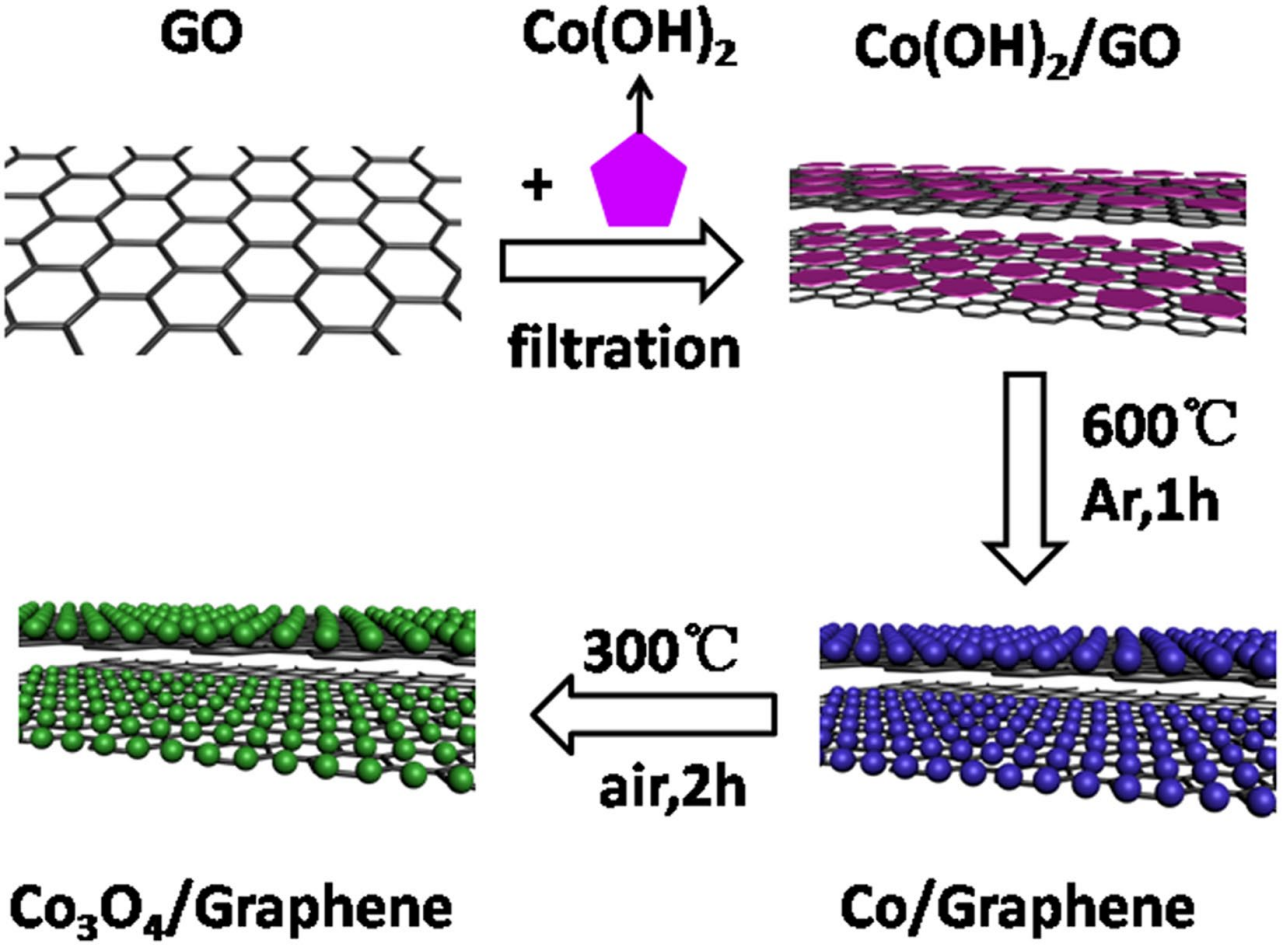
The fabrication process of the self-supporting Co_3_O_4_/GS hybrid film.

Figure 1 displayed the synthesis process of Co_3_O_4_/GS hybrid films. The surface of GO, functionalized with oxygen-containing functional groups^17^, was measured to be a negatively charged surface (zeta potential: −60 mV) in this study. Co(OH)_2_ colloid, which was tested to be with a zeta potential of +22 mV, was a positively charged dispersion with high stability. Learning from colloid science, there are strong electrostatic attractive interactions between two colloid with opposite charges. As a result, a flocculent solution was produced immediately when mixing GO colloid with Co(OH)_2_ colloid, indicating an excellent self-assembly of the two dispersions driven by the strong electrostatic attractive interaction^18^. Afterwards, the flocculent solution can be easily vacuum-filtrated to construct a self-supporting Co(OH)_2_/GO hybrid film. Further two-step heat treatments will lead to the construction of a porous and free-standing Co_3_O_4_/GS hybrid film which can be used as anode electrode directly.

**Figure 2:**
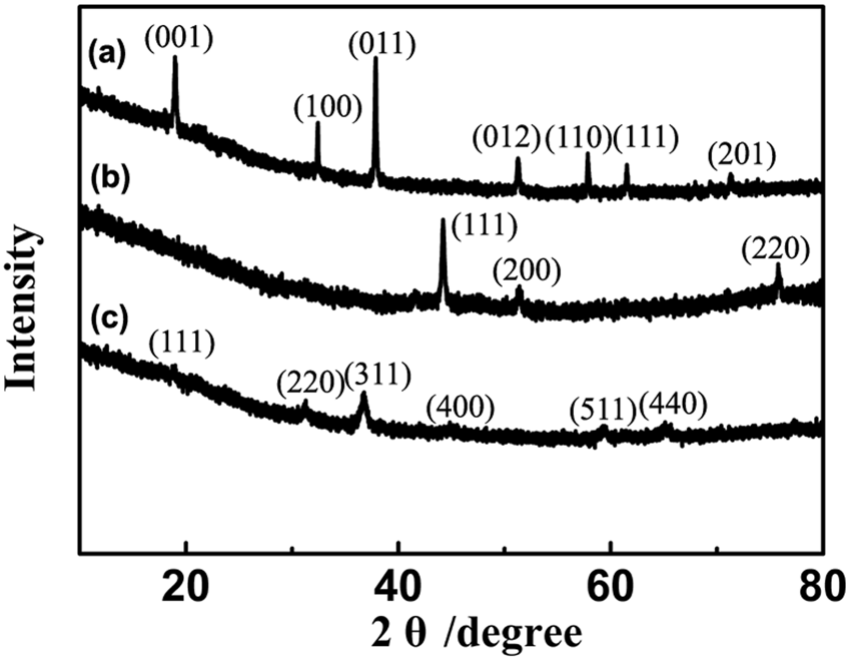
XRD patterns of the hybrid films: (**a**) Co(OH)_2_/GO, (**b**) Co/GS, (**c**) Co_3_O_4_/GS hybrid films.

XRD was employed to characterize the phase transformation of Co_3_O_4_ during preparation procedures. As shown in XRD pattern of Co(OH)_2_/GO hybrid films (Figure 2a), the well-defined diffraction peaks can be successfully indexed to hexagonal β-Co(OH)_2_ (JCPDS no. 74–1057). No obvious peaks of GO were detected, because graphene oxide sheets were highly separated by Co(OH)_2_ nanosheets. After annealed in Ar atmosphere at 600 °C for 1 h, Co(OH)_2_/GO was first converted to Co/GS as shown in Figure 2b, in which three main XRD peaks can be well assigned to Co phase (JCPDS no. 15–0806). Noteworthy, there are also no peaks of GS been detected, indicating graphene sheets were well dispersed without any aggregation. After the second step oxidation, clearly, Co phase was successfully transformed to face-centered cubic Co_3_O_4_ (Figure 2c, JCPDS no. 42–1467). No impurity peaks were observed, which manifests that the Co(OH)_2_ was completely transformed to pure Co_3_O_4_ via a two-step heat treatments. The absence of peaks for GS suggest that graphene maintained the highly dispersibility and the restacking of graphene was well controlled during the whole preparation.

**Figure 3:**
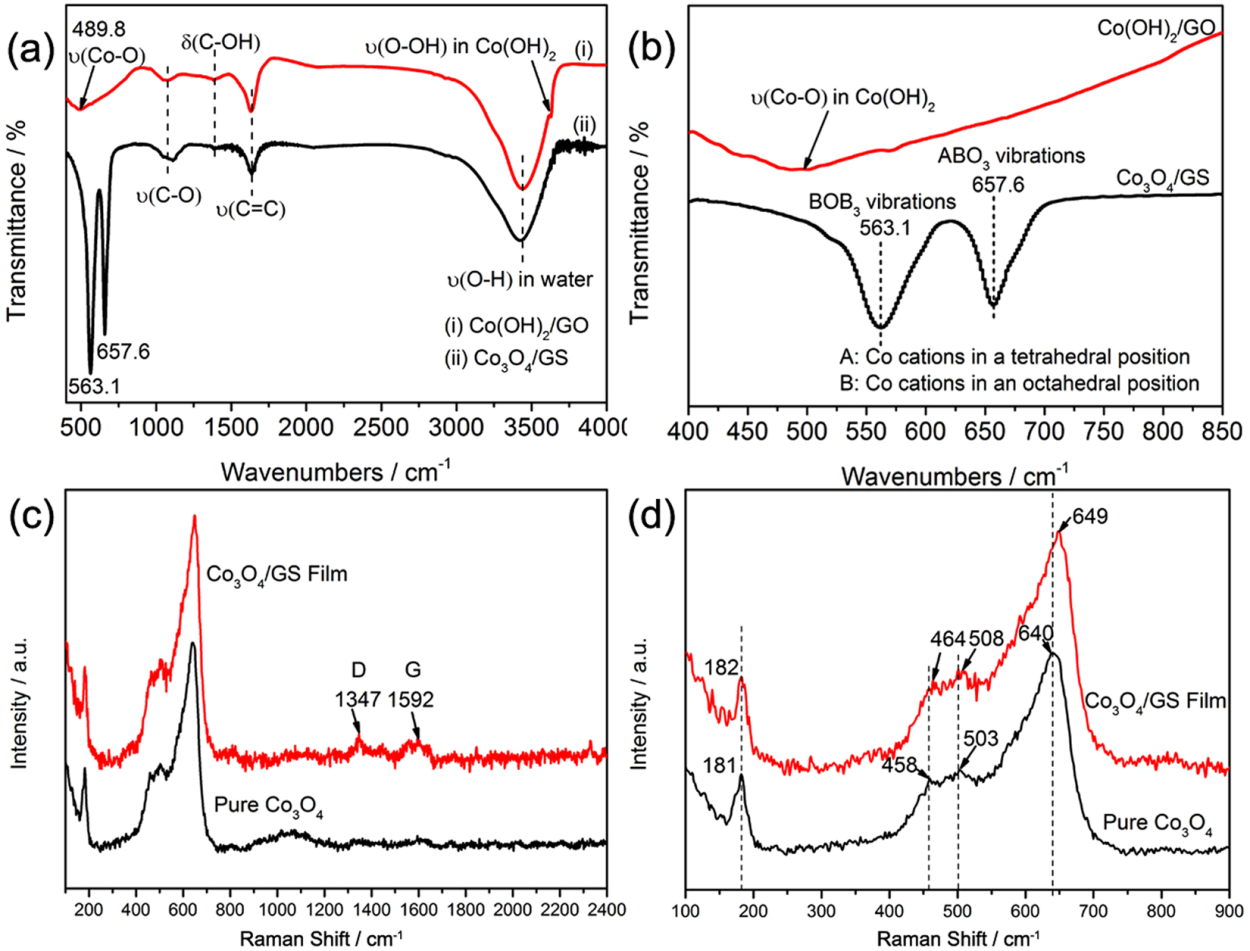
(**a**) FTIR spectra and (**b**) the enlarged spectra of Co(OH)_2_/GO and Co_3_O_4_/GS hybrid films. (**c**) Raman spectra and (**d**) the enlarged Raman spectra of pure Co_3_O_4_ and Co_3_O_4_/GS hybrid films.

The chemical changes from precursors to Co_3_O_4_/GS hybrid films were analyzed by FTIR spectra. As displayed in the FTIR spectrum of Co(OH)_2_/GO (Figure 3a), the visible peaks at about 3437, 1640 and 1077 cm^−1^ correspond to the stretching vibration of -OH in water, stretching vibration of C=C and C-O, respectively, while the peak locating at 1390 cm^−1^ represents the bending vibration of C-OH^19,20^. Apart from these characteristic peaks of oxygen functional groups originating from GO^19,20^, the spectrum of Co(OH)_2_/GO also exhibited stretching vibrations bands of Co-O bond centered at 489.8 cm^−1^ and O-OH bond in Co(OH)_2_ (~3631 cm^−1^)^21,22^. After thermal treatment, the intensities of peaks belonging to oxygen-containing functional groups significantly decreased for Co_3_O_4_/GS, suggesting the effective reduction of GO due to the thermal decomposition of these oxygen-containing functional group^23,24^. Meanwhile, the characteristic peak of υ(O-OH) in Co(OH)_2_ disappeared, instead with two distinctive peaks appeared at 563.1 and 657.6 cm^−1^, respectively. The first band υ1 at 563.1 cm^−1^corresponds to the BOB_3_ vibrations in the spinel lattice, in which B is associated with the Co cations in an octahedral position, i.e. Co^3+^ ions^4,8,10,25^. The second bands υ2 at 657.6 cm^−1^ is resulted from the ABO_3_ vibrations (A: the metal ions in a tetrahedral position). These two stretching band can be well assigned to the characteristic peaks of Co_3_O_4_, further confirming the successful formation of Co_3_O_4_.

Figures 3c and 3d depicted the Raman spectrum of Co_3_O_4_ and Co_3_O_4_/GS composites. The three specific peaks at 458, 503 and 640 cm^−1^ can be assigned to E_g_, F_2g_^1^ and A_g_^1^ vibration modes of pristine Co_3_O_4_, respectively^26,27^. While the spectrum of Co_3_O_4_/GS displayed two distinctive peaks at 1347 and 1592 cm^−1^ apart from those for Co_3_O_4_, corresponding to the D and G band of graphene^28,29^, respectively. This further proves the successful preparation of Co_3_O_4_/GS composites, in accordance with the XRD and FTIR results. As shown in Figure 3d, the enlarged Raman spectra, the characteristic peaks representing Co_3_O_4_ adsorption bands in the hybrid film showed significant blue shifts compared with those of pure Co_3_O_4_. This suggests that there are significant interfacial interactions between Co_3_O_4_ and graphene within Co_3_O_4_/GS hybrid films, which would greatly influence the interfacial lithium ion and electron transport.

**Figure 4:**
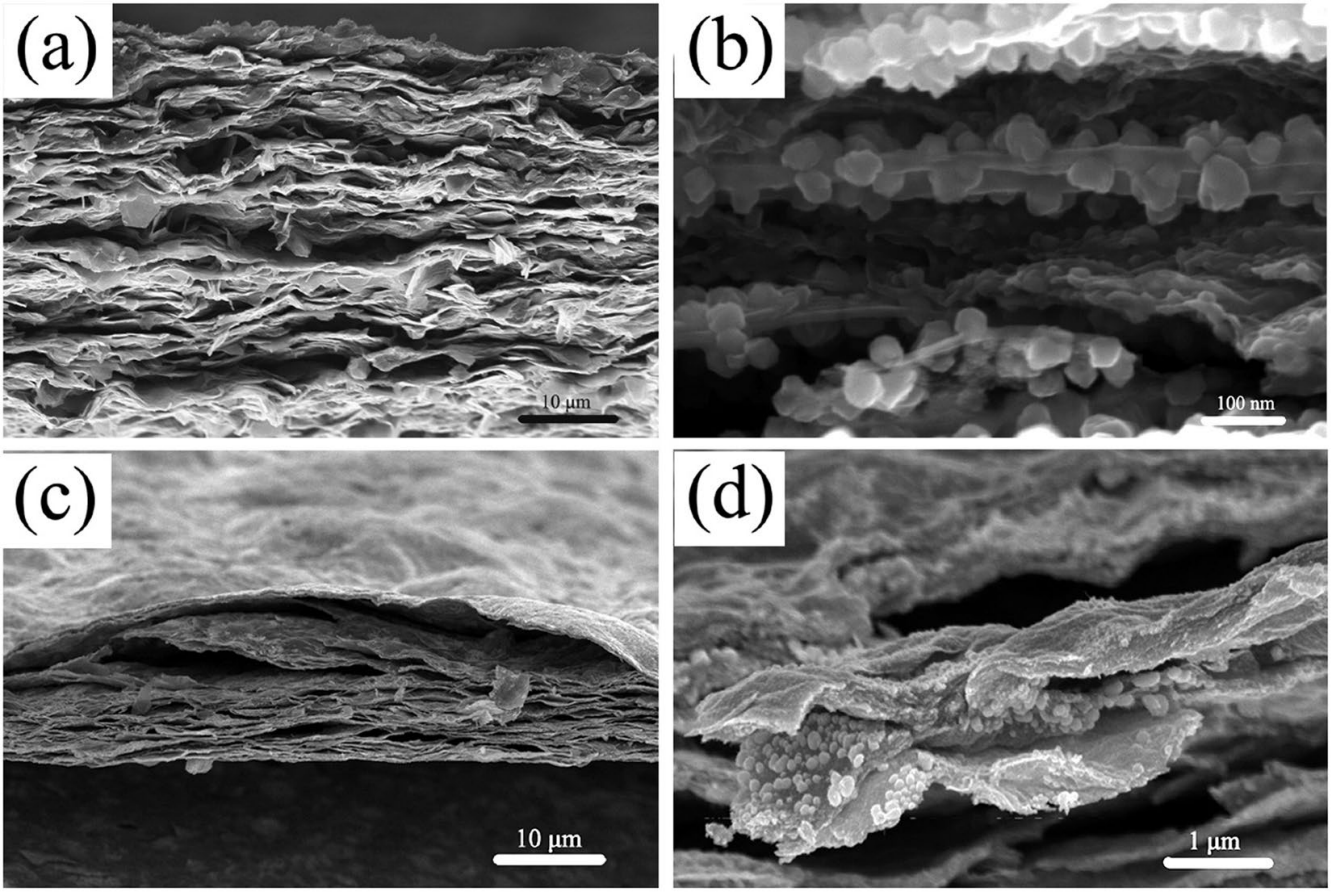
Cross-sectional SEM images: (**a**,**b**) Co/GS film; (**c**,**d**) Co_3_O_4_/GS film.

The microstructures of the hybrid films were illustrated by SEM. The as synthesized Co(OH)_2_ shows a sheet-like morphology and will be well dispersed on the matrix of GO via electrostatic attractive force (Figure S1)^22^. After vacuum-filtration, a free-standing hybrid film with a compact layer-by-layer structure was obtained subsequently(shown in Figure S2). Interestingly, after annealing at 600 °C in Ar for 1 h, Co(OH)_2_ nanosheets firstly decomposed and then *in-situ* reduced to cobalt nanoparticles instead of nanosheets (Figure 4a and 4b). More importantly, Co nanoparticles tightly and uniformly decorated on both surface sides of GS, suggesting strong interfacial interactions, presumably covalent bond, have formed between them. The cobalt was further *in-situ* transformed to Co_3_O_4_ nanoparticles with size of 40~60 nm via oxidation treatment, bringing in a self-supporting film with a porous interleaved structure (Figure 4c and 4d). The diameter of the hybrid film was about 38 mm (Figure S3), with a thickness of ~10μm (Figure 4c). The novel interleaved porous structure of Co_3_O_4_/GS hybrid film will greatly facilitate fast Li-ion diffusion within the electrode, and also supply abundant buffer space to allow the volume expansion of Co_3_O_4_ during charge/discharge processes.

## Discussion

**Figure 5:**
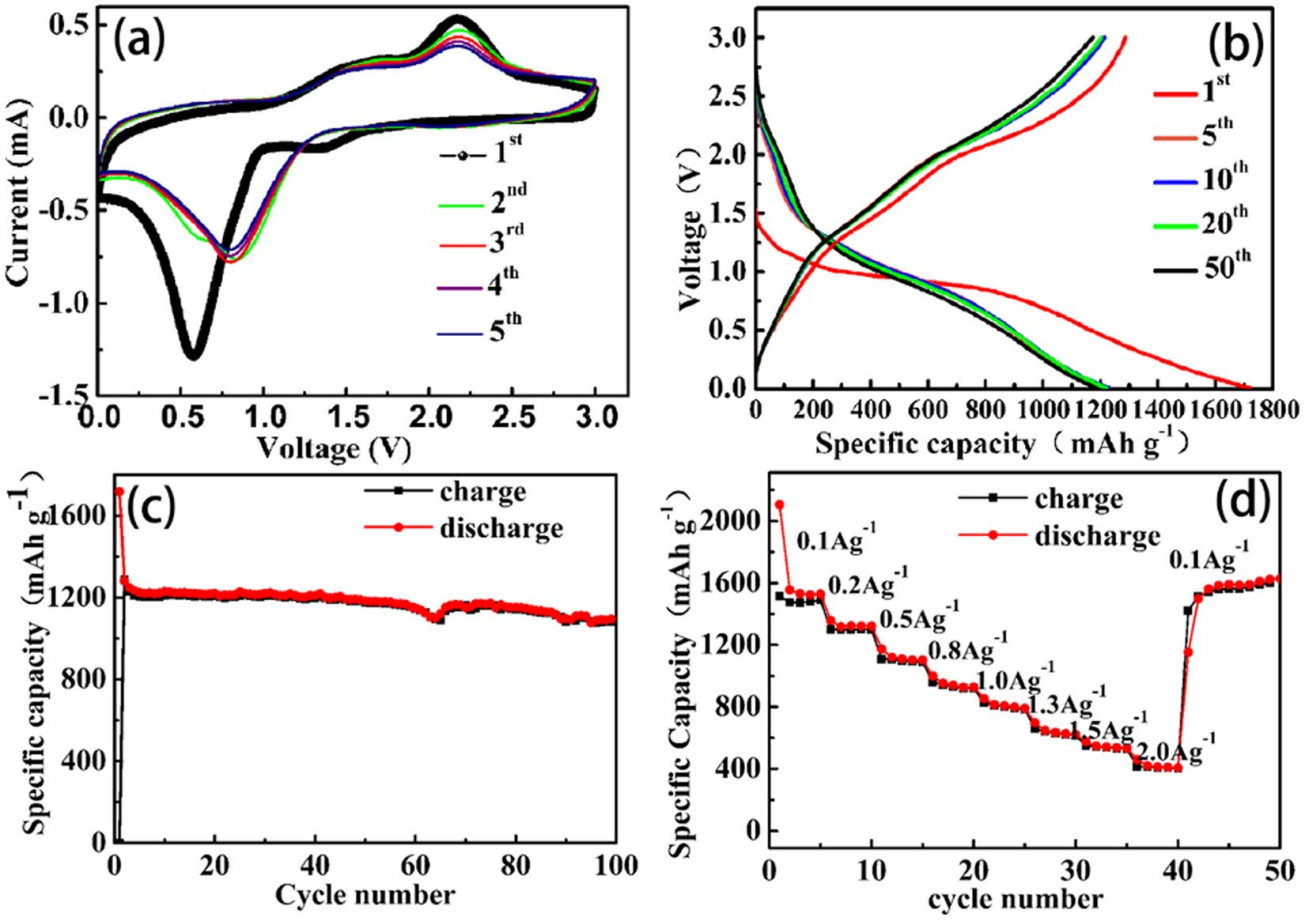
(**a**) Cyclic voltammograms of Co_3_O_4_/GS at 0.5 mVs^−1^; (**b**) Charge/discharge voltage profiles and (**c**) Cycling performance of Co_3_O_4_/GS at a current density of 0.2 A g^−1^; (**d**) Rate capability of Co_3_O_4_/GS hybrid film.

The hybrid film was directly used as electrodes, in which no polymer binder or carbon additives was added. Figure 5 illustrates the electrochemical properties of the binder-free Co_3_O_4_/GS hybrid electrode. As shown in the CV curves, a strong cathodic peak at about 0.6 V is observed in the first discharge process, corresponding to the multi-step electrochemical reduction between Li ions and Co_3_O_4_. Two anodic peaks appeared at ~1.5 and 2.3 V, owing to the oxidation of Co atoms and in consistent with the reported literatures^30,31^. The reactions can be described as:

1 $${{\rm{Co}}}_{3}{{\rm{O}}}_{4}+8{{\rm{Li}}}^{+}+8{{\rm{e}}}^{-}\to 3{\rm{Co}}+4{{\rm{Li}}}_{2}{\rm{O}}$$

2 $${\rm{Co}}+{{\rm{Li}}}_{2}{\rm{O}}\to 2{{\rm{Li}}}^{+}+{\rm{CoO}}+2{{\rm{e}}}^{-}$$

In the subsequent cycles, the cathodic peaks shifted to ~0.75 V, showing a tendency toward stabilization. The anodic peaks were almost the same and overlapped together, indicating the good electrochemical reversibility of the hybrid electrode.

The lithium-storage performance of the binder-free Co_3_O_4_/GS film was characterized by galvanostatic charge/discharge at 0.2 A g^−1^ (Figure 5b). In the discharge curve of first cycle, the potential quickly falls to a long potential plateau at ~1.5 V and then gradually decreased to 0.01 V (the cutoff voltage), which is in analogy with the behavior of Co_3_O_4_ anode. The first discharge/charge capacities reached 1718.5 and 1287.7 mAh g^−1^, respectively, corresponding to the initial coulombic efficiency (CE) of 74.9%, which was comparable to that of Co_3_O_4_/graphene hybrid electrodes reported previously^12,32^. The relative low initial CE value may be ascribed to the inevitable formation of a solid electrolyte interface (SEI) film over the electrode during the charge/discharge process, which led to the insufficient release of capacity. A well-established conductive network may help to further increase the CE^32^, such as surface modification^13^, pre-doping lithium metal^33^ and encapsulation^34^. Compared with the theoretical capacity of graphene (372 mAhg^−1^) and Co_3_O_4_ (890 mAh g^−1^), the extra capacity may owe to the formation of SEI film or interfacial Li-ion storage^35,36^. After fifty charge/discharge cycles, the discharge capacity still remains up to 1184.2 mAh g^−1^, demonstrating the good structural stability of the Co_3_O_4_/graphene anode.

Figure 5c highlights the cycling stability of the Co_3_O_4_/GS anode at 0.2 A g^−1^. The hybrid anode retains a reversible capacity of 1095.1 mAhg^−1^ after 100 cycles with the capacity retention of 85.5%. The rate capacity of the self-supported Co_3_O_4_/GS electrode has also been studied in Figure 5d. The capacities of Co_3_O_4_/GS heterostructures were about 1480, 1300, 1110, 920, 800, 620, 530 and 410 mAh g^−1^ at the current densities of 0.1, 0.2, 0.5, 0.8, 1.0, 1.3, 1.5 and 2.0 A g^−1^, respectively, manifesting an excellent rate capability. Importantly, the capacity re-increased to 1601.2 mAh g^−1^ when the current density returns back to 0.1 A g^−1^, further demonstrating the good reversibility. Compared with the capacities and cycling performance of other Co_3_O_4_-based anode materials reported previously (shown in Table 1), the constructedCo_3_O_4_/GS anode exhibited higher capacity and better stability than Co_3_O_4_/nitrogen doped graphene^13^, graphene/Co_3_O_4_ nanotubes^34^, plasma treated Co_3_O_4_/Graphene^37^, Co_3_O_4_/CC@graphene^38^, Co_3_O_4_/graphene sheet on sheet^39^ and so on. For the as-prepared Co_3_O_4_/GS hybrid film, the strong interfacial interactions between Co_3_O_4_ and GS can greatly facilitate the interfacial charges transportations, which is beneficial to enhance the lithium-storage capacity at high current densities. In addition, more active sites were achieved owing to the small particle size of Co_3_O_4_ and porous interleaved structure, accounting for the enhanced capacity. The flexible graphene substrate and porous structure, can provide buffer space for volume change of Co_3_O_4_ nanoparticles and effectively prevent their aggregation, which contributes to the excellent cycling stability. Overall, the Co_3_O_4_/GS hybrid film delivered superior electrochemical performance benefitting from the synergistic effects of strong interfacial interactions between Co_3_O_4_ and graphene, small particle size of Co_3_O_4_, the interleaved porous structure and binder-free electrode nature, suggesting the superiority of using the hybrid films as an anode material for LIBs. Moreover, the two-step fabrication process is simple, controllable and low-cost, showing great promise to be used in practical applications

**Table 1:** Comparison of electrochemical performance of anode materials based on Co_3_O_4_ and graphene.

Electrode Material	Capacityretention	Specific capacity (mA h g^−1^)	Reference
Co_3_O_4_/Graphene	85.5% (0.2 A g^−1^, 100^th^ cycle)	800 (1 A g^−1^)	This work
Co_3_O_4_/nitrogen doped graphene	67% (0.1 A g^−1^, 200^th^ cycle)	700 (1 A g^−1^)	Ref.^13^
Sandwich-like Co_3_O_4_/Graphene	85.3% (0.2 A g^−1^,100^th^ cycle)	899.8 (1 A g^−1^)	Ref.^32^
Graphene/Co_3_O_4_ nanotubes	89% (0.1 A g^−1^, 80^th^ cycle)	~600 (0.8 A g^−1^)	Ref.^34^
Plasma-treated Co_3_O_4_/graphene	75% (0.125 A g^−1^, 50^th^ cycle)	400 (0.95 A g^−1^)	Ref.^37^
Co_3_O_4_/CC@Graphene	39% (0.1 A g^−1^, 100^th^ cycle)	469(0.05 A g^−1^)	Ref.^38^
Co_3_O_4_–graphene sheet-on-sheet	76% (0.1 A g^−1^, 50^th^ cycle)	~400 (0.8 A g^−1^)	Ref.^39^

## Conclusion

In summary, a vacuum filtration procedure combined with two-step heat treatment has been developed to construct self-supporting Co_3_O_4_/GS hybrid films, with Co_3_O_4_ nanoparticles (40–60 nm) uniformly and tightly decorated on both surface of GS. The formed porous and interleaved microstructure exhibits critical characters as desired anode materials for LIBs, such as strong interfacial interactions, short transport length for Li-ion, and sufficient space for stress relaxation. Consequently, the constructed Co_3_O_4_/GS hybrid film as binder-free electrode exhibited a high capacity of 1287.7 mAh g^−1^ at 0.2 A g^−1^, good cycling stability (capacity retention of 85.5% after 100 cycles at 0.2 A g^−1^) and superior rate capability, making the hybrid films competitive as LIBs anode materials.

## Materials and Methods

β-Co(OH)_2_ was synthesized based on previous report and further diluted to a homogeneous dispersion (0.5 mg mL^−1^)^40,41^. Graphene oxide (GO) was prepared using a modified Hummers method with natural graphite as raw materials^42^, which was also diluted to 0.5 mg mL^−1^ for further use.

For preparation of Co_3_O_4_/GS film, the β-Co(OH)_2_ and GO dispersion were mixed together by sonication; and then vacuum filtered to form a self-supporting Co(OH)_2_/GO hybrid film. Afterwards, two-step heat treatment approach was carried out: First, the obtained Co(OH)_2_/GO film was heat-treated in Ar at 600 °C for 1 h to produce Co/GS. Second, Co/GS was further oxidized to Co_3_O_4_/GS by being calcined in air (300 °C, 2 h).

### Materials Characterizations

The morphologies and microstructures of the samples were characterized via field-emission scanning electron microscopy(FE-SEMJSM-6700F). The crystalline phase of the materials was analyzed from X-ray diffraction measurements (Rigaku D/max 2550 V diffractometer). Raman spectroscopy was conducted on DXR Raman Microscope (Thermal Scientific Corporation, USA, wavelength 532 nm). Fourier transform infrared spectroscopy (FTIR) were examined on Nicolet 7000-C.

### Electrochemical Measurements

In this paper, the as-synthesized self-supporting Co_3_O_4_/GS hybrid films were directly used as an electrode without adding any binder or additive. The cell was assembled in glove box, and Li foil was adopted as the counter electrode. The 1 M LiPF_6_ in a mixture of ethylene carbonate (EC)/dimethyl carbonate (DMC)(50:50, by volume) was used as electrolyte. The electrochemical properties were measured using a CT2001 battery tester at room temperature. Cyclic voltammetry (CV) was performed via electrochemical workstation (CHI760E) at a scan rate of 0.5 mV s^−1^ within a voltage range of 0–3.0 V. The mass loading was about 0.8 mg for each Co_3_O_4_/GS electrode.

## Electronic supplementary material

Supplementary Information
