# Supplementary material for: Self-supporting Co3O4/Graphene Hybrid Films as Binder-free Anode Materials for Lithium Ion Batteries
Source: Sci Rep. 2018 Feb 16;8:3182. doi: 10.1038/s41598-018-21436-4 (PMC5816628; doi:10.1038/s41598-018-21436-4)
Supplement: Supplementary file 1 — Supplementary Information [file 41598_2018_21436_MOESM1_ESM.doc]

**Supplementary Information**

**Self-supporting Co_3_O_4_/Graphene Hybrid Films as Binder-free Anode Materials for Lithium Ion Batteries**

Shouling Wang^c^, Ronghua Wang*^a^**, Jie Chang^c^, Ning Hu*^b^*, and Chaohe Xu *^b,d^**

*^a^ College of Materials Science and Engineering, Chongqing University, Chongqing, 400044, China*

*^b^ College of Aerospace Engineering, and The State Key Laboratory of Mechanical Transmissions, Chongqing University, Chongqing 400044, China*

*^c^* *School of Chemistry and Materials Engineering, Chizhou University, Chizhou, 247000, China*

*^d^Key Laboratory of Low-grade Energy Utilization Technologies and Systems of the Ministry of Education of China, Chongqing, 400044, China*

* Corresponding author

E-mail: [wangrh@cqu.edu.cn](mailto:wangrh@cqu.edu.cn); [xche@cqu.edu.cn](mailto:xche@cqu.edu.cn)


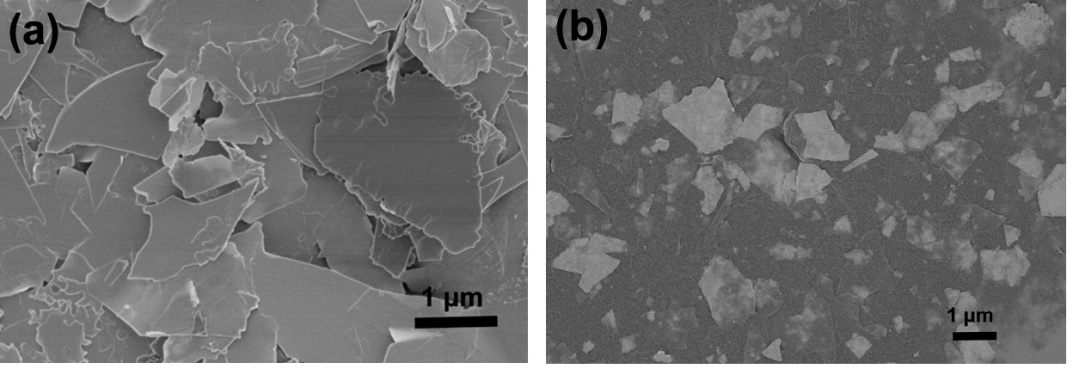


**Fig. S1** SEM images of (a) pure Co(OH)_2_ and (b) Co(OH)_2_/GO dispersion.


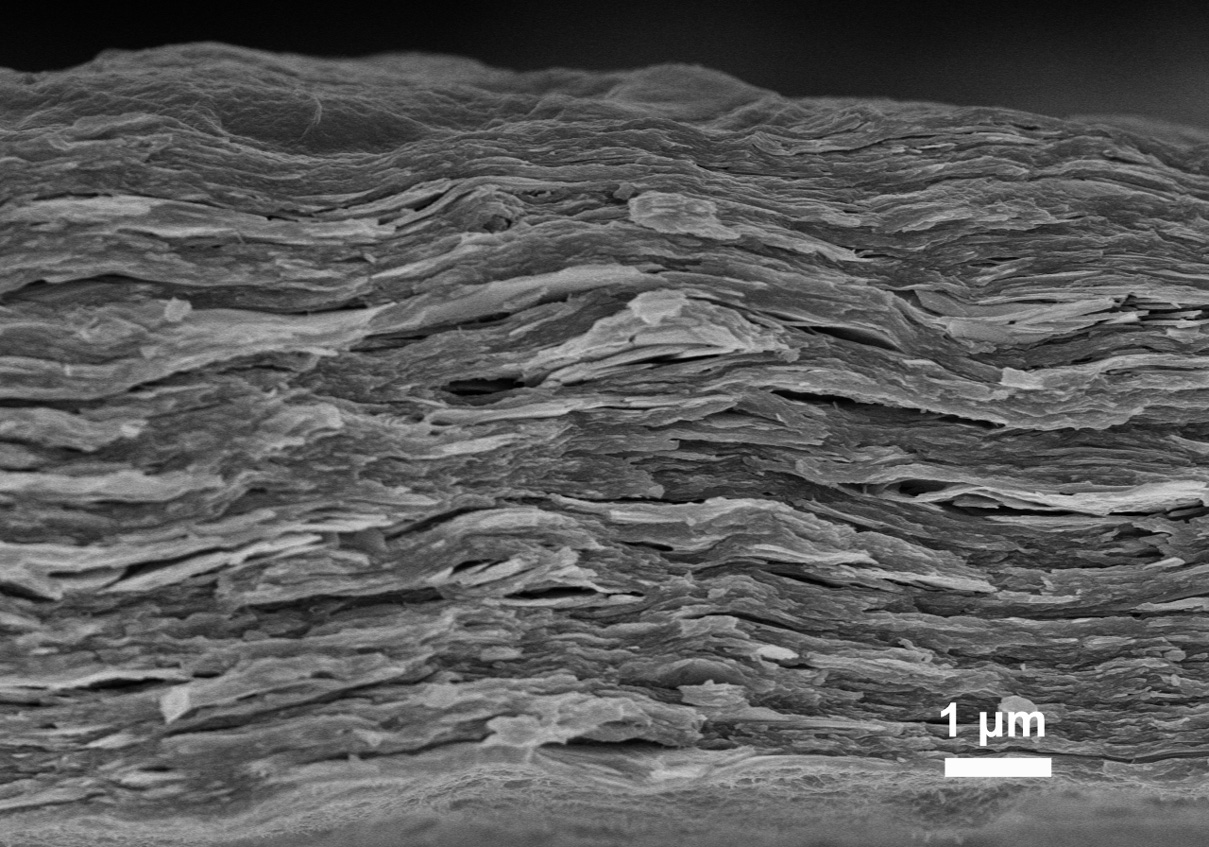


**Fig. S2** Cross-sectional SEM images of Co(OH)_2_/GO hybrid films.


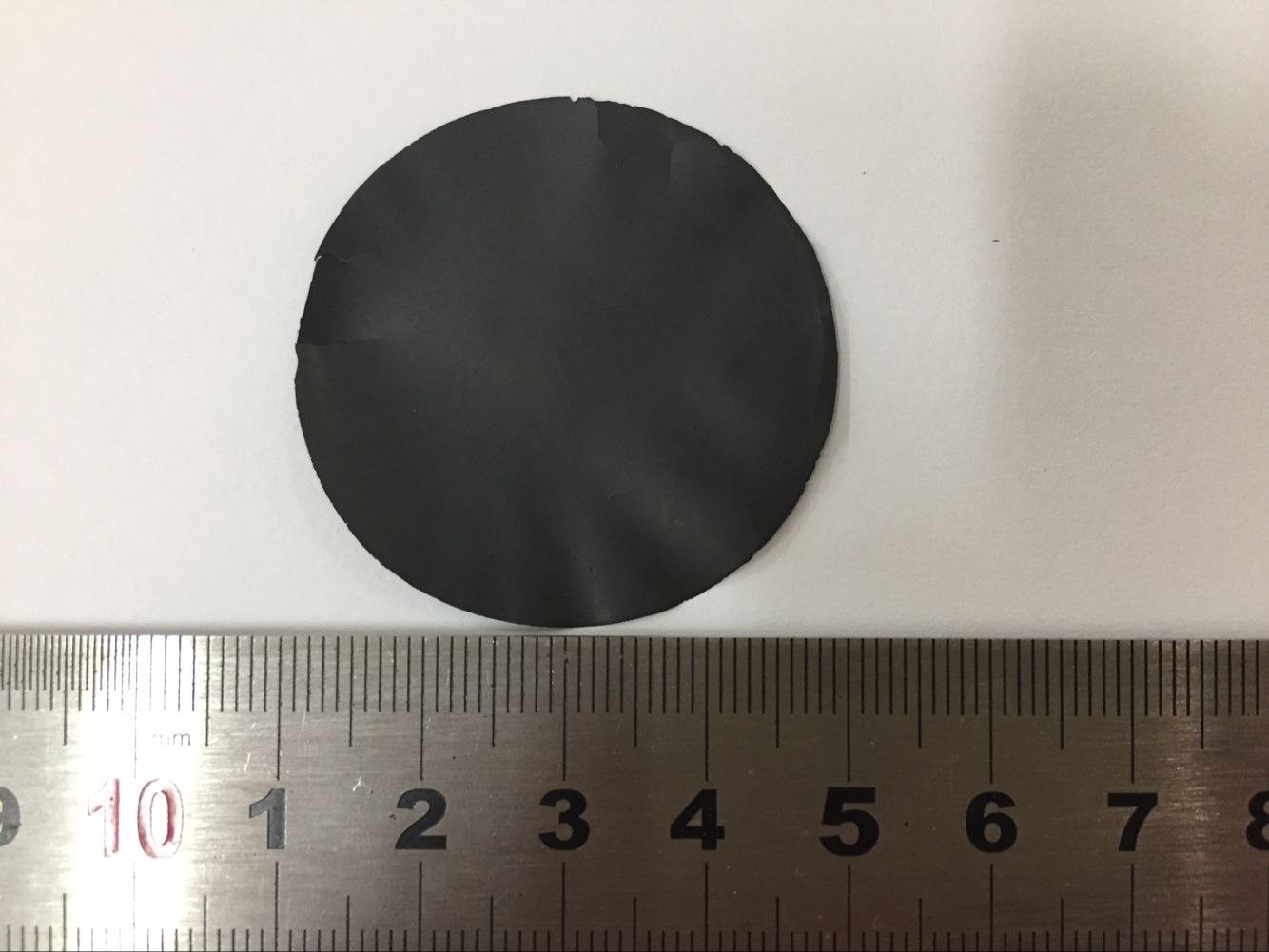


**Fig. S3** A digital photograph of the Co_3_O_4/_graphene hybrid film.
